# Psychological capital has a positive correlation with humanistic care ability among nurses

**DOI:** 10.3389/fpsyg.2022.955627

**Published:** 2022-09-16

**Authors:** Xiaohong Liu, Cuiping Li, Xiaoting Yan, Bingqing Shi

**Affiliations:** ^1^School of Medicine, Taizhou University, Taizhou, China; ^2^Central Sterile Supply Department, Taizhou Central Hospital (Taizhou University Hospital), Taizhou, China

**Keywords:** nurse, psychological capital, humanistic care ability, self-efficacy, positive psychology, training

## Abstract

**Objective:**

With the improvement in health awareness, humanistic care ability of nurses has become a focus of public attention. The aim of the study was to confirm the relationship between psychological capital and humanistic care ability of nurses, and to provide suggestions on improving the humanistic care ability of nurses.

**Methods:**

A cross-section survey was conducted. Three hundred thirty-nine nurses were recruited from a tertiary general hospital in Taizhou, China. Psychological capital and humanistic care ability were measured using a self-reported questionnaire. Correlation analysis and stepwise multiple regression analysis were performed to analyze the correlation between psychological capital and humanistic care ability.

**Results:**

The psychological capital and humanistic care ability scores were 91.57 ± 13.96 and 189.08 ± 20.37, respectively. Differences in psychological capital scores across professional titles (*F* = 4.88, *p* = 0.01), departments (*F* = 3.69, *p* < 0.001), years of work (*F* = 4.68, *p* < 0.001), and marital status (*t* = 3.25, *p* < 0.001) were statistically significant. There were statistical differences for the humanistic care ability scores among nurses based on marital status (*p* = 0.01). The total psychological capital scores and the four sub-dimensions scores were positively correlated with the humanistic care ability scores among nurses. Self-efficacy was the main predictor of nurses’ humanistic care ability.

**Conclusion:**

Psychological capital positively affected the humanistic care ability of nurses. Self-efficacy was the main predictor of humanistic care ability. Nursing managers can formulate strategies from the perspective of positive psychology to improve humanistic care ability of nurses.

## Introduction

Nursing is an art that emphasizes the nature of caring (Yeh and Lee, 2011). Care that is based exclusively on objective knowledge might be unsafe and low quality (Rafii et al., 2021). Humanistic care, one of the core competencies of nurses, is a type of value and attitude that cares about people’s willingness, consciousness, or responsibility, and reflects particular actions (McCance et al., 1999). Nurses should have humanistic qualities to identify what the patients need with effective care plans, and meet these needs (Yanmis et al., 2022). Nevertheless, the humanistic care ability of nurses in China is currently at a level (Sheng et al., 2018) that cannot meet the requirements of high-quality nursing services (Gao et al., 2021). Technology continues to be valued over humanistic values, especially in resource-constrained work environments (Bu et al., 2021).

Humanistic caring is integrated into nursing care (Letourneau et al., 2021). Humanistic caring is not simply to meet the clinical needs of the patients, it requires medical professionals to pursue excellence in many aspects of caring, especially with the spiritual, philosophical, ethical and moral dimensions (Calle et al., 2017). The nurse and patient, organizational environment (Mohamadi Asl et al., 2022), psychological characteristics (Kovner et al., 2006) and society give rise to a set of intertwined characteristics which influence the realization of humanistic caring. Nurses with higher humanistic care ability are able to provide effective clinical practice and offer high quality of care in hospitals, which contributes to improvement in metrics such as higher patients’ satisfaction, less work pressure and more harmonious doctor-patient relationship (Raja et al., 2015). Nursing managers have adopted a variety of strategies to improve humanistic care ability, such as improving nurses’ working conditions (Letourneau et al., 2021), conducting standardized training (Yan et al., 2022) and providing continuous learning (Wang et al., 2020). Nevertheless, due to the increasing workload of clinical nurses and the lack of nursing human resources, the implementation of humanistic care has been hindered to some extent (Lai et al., 2022).

Research has shown that a lack of psychological resources and solidified personality traits are the leading reasons for the low level of humanistic care ability (Li et al., 2017). Therefore, it is necessary to explore strategies to increase psychological resources in order to improve humanistic care ability.

Psychological capital is defined as an individual’s positive mental state during his/her growth and development (Luthans et al., 2007b). As a positive psychological characteristic, psychological capital strengthens an individual’s abilities and increases the resources to overcome difficult situations and achieve success (Guo et al., 2021). Individuals may better cope with stress if they have more psychological capital (Elliott and Fry, 2021). Positive psychological capital is an important manifestation of individual physical and mental health, which can effectively strengthen job involvement and subjective well-being, and inhibit silent behavior (Kaya and Eskin Bacaksiz, 2021). Positive psychological capital increases organizational commitment and decreases job burnout, and also reduces symptoms of anxiety among female nurses (Zhou et al., 2018). Positive psychological capital influences work-related performance of nurses (Dwyer et al., 2019; An et al., 2020).

To advance our knowledge of the relationship between psychological capital and humanistic care ability, we draw on Watson’s theory of human caring (Smith and Parker, 2015) and psychological capital theory (Luthans et al., 2004). Watson puts forward ten carative factors as a framework for providing a format and focus for nursing phenomena. Instillation of faith-hope and promotion and acceptance of the expression of positive and negative feelings are the two factors. Studies (Antonini et al., 2021; Goral Turkcu and Ozkan, 2021) have shown that applying Watson’s theory of human caring to the practice of humanistic care can improve nurse’s self-confidence, time management, work engagement, and benevolent behaviors. Positive psychological capital is the concept of positive organizational behavior based on psychology, which emphasizes the importance of positive psychological values. Positive psychological capital is composed of four independent components: self-efficacy; optimism; hope; and resilience (Luthans et al., 2004). Psychological capital can affect the attitudes and behaviors of individuals (Kaya and Eskin Bacaksiz, 2021). Positive psychological capital is predicted to affect person-centered care competence, which refers to a humanistic care approach by reducing negative aspects and amplifying positive aspects (Kim, 2022). The relationship between psychological capital and humanistic care ability is becoming the focus of research.

Studies involving the relationship between psychological capital and humanistic care are mostly focused on nursing students. Studies have shown that humanistic care ability was positively correlated with psychological capital among nursing students (Jiang et al., 2017). Positive psychological capital and adaptive emotion expression had multiple mediating roles between individual social capital and humanistic care ability in nursing students (Shen et al., 2020). Lai et al. (2022) studied the association between positive mental character and humanistic care ability in nursing students. However, there is little information about the relationship between psychological capital and humanistic care ability among nurses. It is important to explore the relationship between psychological capital and humanistic care ability of nurses.

## Materials and methods

### Participants and procedure

This is a cross-section study. In order to ensure that the samples were representative and comparable, the work department and years of employment were set as the stratified sampling basis. Research subjects were selected from internal medicine (including gastroenterology, endocrinology, respiratory medicine, cardiology, hematology, nephrology, neurology, radiation therapy, geriatrics, and a combination of traditional Chinese and Western medicine), surgery (including breast surgery, gastrointestinal surgery, urology, thoracic surgery, orthopedics, neurosurgery, and liver and gallbladder surgery), obstetrics and gynecology, pediatrics, emergency medicine and the intensive care units (ICUs). Sample size was selected in proportion to the number of nurses in the department. From January to March 2020, with the support of nursing personnel department of a tertiary Grade A general hospital in Taizhou (a city on the east coast of China), 370 questionnaires were distributed to all departments and 339 valid questionnaires were returned (effective rate = 91.62%); the samples with missing and incomplete answers were excluded. Before filling out the questionnaire, the researchers explained the purpose, content, and significance of the research to the subjects using standardized guidelines. Informed consent was obtained from all subjects. All personal information was deidentified.

All of the nurses in this study have worked in the hospital for >6 months. The descriptive statistical results of the sample were as follows: average age = 30.50 ± 6.32 years; 326 (96.17%) females; and 13 (3.83%) males. Two hundred twelve (62.54%) were primary nurses, 113 (33.33%) were intermediate nurses, and 14 (4.13%) were senior nurses. Two (0.59%) nurses graduated from technical secondary school, 93 (27.43%) graduated from junior college, 243 (71.68%) graduated from undergraduate programs, and one (0.3%) graduated from a graduate program. One hundred thirty-six (40.12%) were unmarried and 203 (59.88%) were married.

### Measures

#### Psychological capital questionnaire

Psychological capital in nurse participants was evaluated by the psychological capital questionnaire [PCQ] (Luthans et al., 2007a). The Chinese version of the PCQ, which was developed by Luo and He (2010), contains 20 items in four dimensions: self-efficacy (six items); hope (six items); resilience (five items); and optimism (three items). Self-efficacy is the knowledge and confidence to carry out specific tasks. Hope is an ability to persevere with goals successfully. Resilience is characterized by the ability to succeed, learn, and become stronger in the face of challenges. Optimism is consciously cultivating a positive attribution to succeed (Luthans and Youssef-Morgan, 2017). Each item was measured using a 6-point Likert scale, ranging from 1 (strongly disagree) to 6 (strongly agree). The total score ranged from 20 to 120. The higher the score, the higher the psychological capital. The validation study showed that the questionnaire demonstrated adequate validity and reliability. The Cronbach alpha ranged from 0.718 to 0.923.

#### Caring ability inventory

Humanistic care ability is the core of nursing (Woods, 2011). The Caring Ability Inventory (CAI) designed by Nkongho (1990) is one of the most well-recognized and widely-used instruments to evaluate the humanistic care ability (Ma et al., 2022). The CAI has three dimensions that included a knowing dimension (14 items), a courage dimension (13 items), and a patience dimension (10 items). Knowing refers to how well you know yourself, others, and your surroundings. Courage refers to the ability to handle unknown situations. Patience refers to endurance and toughness (Ma et al., 2022). Each item was measured using a 7-point Likert scale, ranging from 1 (completely opposed) to 7 (fully agree). The CAI total score was 37 ~ 259 (knowing, 14–98; courage, 13–91; and patience, 10–70). The higher the total score, the stronger the caring ability. A total score < 203.1 was considered low humanistic care ability, a score from 203.1–220.3 was considered medium humanistic care ability, and a score > 220.3 was considered high humanistic care ability. The scale has good reliability with a Cronbach alpha of 0.832. Cronbach alpha coefficients of knowing, courage, and patience were 0.785, 0.720, and 0.748, respectively.

### Data analysis

SPSS20.0 was used for data processing and statistical analysis, which was performed using a *t*-test, analysis of variance, correlation analysis, and multiple stepwise regression analysis. Measurement data were described by the mean ± the standard deviation.

## Results

### Current status of psychological capital and humanistic care ability of nurses

The psychological capital scores of 339 nurses were 91.57 ± 13.96. The self-efficacy, hope, resilience, and optimism scores were 27.3 ± 4.66, 27.27 ± 4.36, 22.68 ± 4.03, and 14.31 ± 2.72, respectively. The humanistic care ability score of nurses was 189.08 ± 20.37 (<203.1), and the knowing, patience, and courage scores were 77.01 ± 10.80, 59.28 ± 6.93, and 52.79 ± 12.17, respectively, which were all lower than the foreign norm (Watson, 2002; Table 1; *p* < 0.01), indicating that the humanistic care ability of nurses in China was generally low.

**Table 1:** Comparison of clinical nurse CAI scale scores between nurses included in this study and the foreign norm.

Item	*n*	Knowing	Courage	Patience
Score of this survey	339	77.01 ± 10.80	52.79 ± 12.17	59.28 ± 6.93
Foreign norm	1,388	80.22 ± 7.56	68.25 ± 11.57	63.11 ± 5.36
*t*		6.39	21.83	11.09
*P*		<0.01	<0.01	<0.01

### Comparison of psychological capital and humanistic care ability among nurses with different characteristics

Differences in psychological capital scores across professional titles (*F* = 4.88, *p* = 0.01), departments (*F* = 3.69, *p* < 0.001), years of employment (*F* = 4.68, *p* < 0.001), and marital status (*t* = 3.25, *p* < 0.001) were all statistically significant. There were statistical differences for the humanistic care ability scores among nurses based on marital status (*p* = 0.01; Table 2). There were no significant differences in gender, education, growth environment, the only child and religious beliefs in psychological capital or humanistic care ability.

**Table 2:** Comparison of the psychological capital score and humanistic care ability among nurses with different characteristics.

Item	*n* (%)	Psychological capital score	*t*/*F*	*p*	Humanistic care ability score	*t*/*F*	*p*
**Gender**			1.03	0.31		1.01	0.31
Male	13(3.83)	95.46 ± 16.44			194.69 ± 19.19		
Female	326(96.17)	91.41 ± 13.86			188.86 ± 20.41		
**Professional title**			4.88	0.01^*^		2.88	0.06
Primary	212(62.54)	90.01 ± 14.40			187.04 ± 20.87		
Intermediate	113(33.33)	93.49 ± 12.43			192.56 ± 19.17		
Senior	14(4.13)	99.71 ± 15.17			191.93 ± 19.37		
**Department**			3.69	0.00^**^		2.58	0.03
Internal medicine	102(30.09)	94.98 ± 15.04			193.93 ± 21.36		
Surgery	85(25.07)	91.73 ± 11.90			188.39 ± 20.07		
Obstetrics and Gynecology	47(13.86)	88.26 ± 16.01			186.15 ± 17.90		
Pediatric	14(4.13)	92.00 ± 7.26			189.64 ± 21.11		
Emergency	44(12.98)	93.09 ± 12.08			189.73 ± 20.99		
ICU	47(13.86)	85.64 ± 13.96			181.98 ± 18.42		
**Education**			0.86	0.46		0.46	0.71
Technical secondary school	2(0.59)	94 ± 7.07			203 ± 26.87		
Junior college	93(27.43)	89.60 ± 12.95			187.91 ± 19.94		
Undergraduate	243(71.68)	92.30 ± 14.36			189.44 ± 20.57		
Graduate	1(0.3)	93 ± 0			184		
**Years of employment**			4.68	0.00^**^		1.25	0.29
<2 years	93(27.43)	87.92 ± 12.34			187.8 ± 20.78		
2–5 years	48(14.16)	91.35 ± 12.28			186.52 ± 19.63		
6–10 years	86(25.37)	90.29 ± 16.26			187.4 ± 21.16		
11–20 years	98(28.91)	95.12 ± 12.47			192.47 ± 19.45		
>20 years	14(4.13)	95.50 ± 16.99			193.07 ± 20.76		
**Growth environment**			0.59	0.56		1.12	0.33
City	49(14.45)	91.06 ± 14.77			185.06 ± 22.70		
Villages and towns	82(24.19)	93.02 ± 10.95			189.68 ± 20.79		
Country	208(61.36)	91.12 ± 14.83			189.79 ± 19.61		
**Only child**			−0.03	0.98		−0.33	0.74
Yes	45(13.27)	91.51 ± 13.04			188.16 ± 15.96		
No	294(86.73)	91.58 ± 14.12			189.22 ± 20.98		
**Marital status**			−3.25	0.00^**^		−2.74	0.01^*^
Single	136(40.12)	88.60 ± 13.67			185.42 ± 20.934		
Married	203(59.88)	93.56 ± 13.84			191.54 ± 19.655		
**Religious belief**			0.35	0.73		1.05	0.30
Have	43(12.68)	92.26 ± 13.28			192.12 ± 20.97		
No	296(87.32)	91.47 ± 14.08			188.64 ± 20.28		

* *p* < 0.05;

** *p* < 0.01.

The results of multiple comparisons are shown in Tables 3–6. Nurses from obstetrics, pediatric and ICU had lower psychological capital scores. The psychological capital and humanistic care ability scores of married nurses were higher than unmarried nurses.

**Table 3:** Multiple comparisons of psychological capital scores of nurses with different professional title.

(I) Professional title	(J) Professional title	Mean difference (I-J)	Std. error	*p*	95% Confidence interval	
					Lower bound	Upper bound
Primary	Intermediate	−3.48^*^	1.61	0.03	−6.64	−0.31
	Senior	−9.70^*^	3.81	0.01	−17.20	−2.21
Intermediate	Primary	3.48^*^	1.61	0.03	0.31	6.64
	Senior	−6.23	3.91	0.11	−13.92	1.47
Senior	Primary	9.70^*^	3.81	0.01	2.21	17.20
	Intermediate	6.23	3.91	0.11	−1.47	13.92

* The mean difference is significant at the 0.05 level.

**Table 4:** Multiple comparisons of psychological capital scores among nurses from different departments.

(I) Department	(J) Department	Mean difference (I−J)	Std. error	*p*	95% Confidence interval	
					Lower bound	Upper bound
Internal medicine	Surgery	3.25	2.01	0.11	−0.71	7.21
	Obstetrics and gynecology	6.73^*^	2.41	0.01	1.98	11.47
	Pediatric	2.98	3.90	0.45	−4.70	10.66
	Emergency	1.89	2.47	0.45	−2.97	6.75
	ICU	9.34^*^	2.41	0.00	4.59	14.09
Surgery	Internal medicine	−3.25	2.01	0.11	−7.21	0.71
	Obstetrics and gynecology	3.47	2.49	0.16	−1.42	8.37
	Pediatric	−0.27	3.95	0.95	−8.04	7.50
	Emergency	−1.36	2.54	0.59	−6.36	3.64
	ICU	6.09^*^	2.49	0.02	1.19	10.99
Obstetrics and gynecology	Internal medicine	−6.73^*^	2.41	0.01	−11.47	−1.98
	Surgery	−3.47	2.49	0.16	−8.37	1.42
	Pediatric	−3.74	4.17	0.37	−11.95	4.46
	Emergency	−4.84	2.87	0.09	−10.49	0.82
	ICU	2.62	2.82	0.36	−2.94	8.17
Pediatric	Internal medicine	−2.98	3.90	0.45	−10.66	4.70
	Surgery	0.27	3.95	0.95	−7.50	8.04
	Obstetrics and gynecology	3.74	4.17	0.37	−4.46	11.95
	Emergency	−1.09	4.20	0.80	−9.36	7.18
	ICU	6.36	4.17	0.13	−1.84	14.56
Emergency	Internal medicine	−1.89	2.47	0.45	−6.75	2.97
	Surgery	1.36	2.54	0.59	−3.64	6.36
	Obstetrics and gynecology	4.84	2.87	0.09	−0.82	10.49
	Pediatric	1.09	4.20	0.80	−7.18	9.36
	ICU	7.45^*^	2.87	0.01	1.80	13.10
ICU	Internal medicine	−9.34^*^	2.41	0.00	−14.09	−4.59
	Surgery	−6.09^*^	2.49	0.02	−10.99	−1.19
	Obstetrics and gynecology	−2.62	2.82	0.36	−8.17	2.94
	Pediatric	−6.36	4.17	0.13	−14.56	1.84
	Emergency	−7.45^*^	2.87	0.01	−13.10	−1.80

* The mean difference is significant at the 0.05 level.

**Table 5:** Multiple comparisons of psychological capital scores of nurses with different years of employment.

(I) Years of employment	(J) Years of employment	Mean difference (I−J)	Std. error	*p*	95% Confidence interval	
					Lower bound	Upper bound
<2 years	2–5 years	−3.43	2.43	0.16	−8.21	1.35
	6–10 years	−2.37	2.05	0.25	−6.39	1.66
	11–20 years	−7.20^*^	1.98	0.00	−11.09	−3.30
	>20 years	−11.58^*^	3.92	0.00	−19.28	−3.87
2–5 years	<2 years	3.43	2.43	0.16	−1.35	8.21
	6–10 years	1.06	2.46	0.67	−3.78	5.91
	11–20 years	−3.77	2.41	0.12	−8.51	0.97
	>20 years	−8.15	4.15	0.05	−16.31	0.02
6–10 years	<2 years	2.37	2.05	0.25	−1.66	6.39
	2–5 years	−1.06	2.46	0.67	−5.91	3.78
	11–20 years	−4.83^*^	2.02	0.02	−8.81	−0.86
	>20 years	−9.21^*^	3.94	0.02	−16.96	−1.46
11–20 years	<2 years	7.20^*^	1.98	0.00	3.30	11.09
	2–5 years	3.77	2.41	0.12	−0.97	8.51
	6–10 years	4.83^*^	2.02	0.02	0.86	8.81
	>20 years	−4.38	3.91	0.26	−12.06	3.31
>20 years	<2 years	11.58^*^	3.92	0.00	3.87	19.28
	2–5 years	8.15	4.15	0.05	−0.02	16.31
	6–10 years	9.21^*^	3.94	0.02	1.46	16.96
	11–20 years	4.38	3.91	0.26	−3.31	12.06

* The mean difference is significant at the 0.05 level.

**Table 6:** Multiple comparisons of humanistic care ability scores among nurses from different departments.

(I) Department	(J) Departments	Mean difference (I-J)	Std. error	*p*	95% Confidence interval	
					Lower bound	Upper bound
Internal medicine	Surgery	5.54	2.96	0.06	−0.27	11.36
	Obstetrics and gynecology	7.78^*^	3.55	0.03	0.80	14.77
	Pediatric	4.29	5.74	0.46	−7.00	15.58
	Emergency	4.20	3.63	0.25	−2.94	11.35
	ICU	11.95^*^	3.55	0.00	4.97	18.94
Surgery	Internal medicine	−5.54	2.96	0.06	−11.36	0.27
	Obstetrics and gynecology	2.24	3.66	0.54	−4.96	9.44
	Pediatric	−1.25	5.81	0.83	−12.68	10.17
	Emergency	−1.34	3.74	0.72	−8.70	6.02
	ICU	6.41	3.66	0.08	−0.79	13.61
Obstetrics and gynecology	Internal medicine	−7.78^*^	3.55	0.03	−14.77	−0.80
	Surgery	−2.24	3.66	0.54	−9.44	4.96
	Pediatric	−3.49	6.13	0.57	−15.55	8.57
	Emergency	−3.58	4.22	0.40	−11.89	4.73
	ICU	4.17	4.15	0.32	−4.00	12.34
Pediatric	Internal medicine	−4.29	5.74	0.46	−15.58	7.00
	Surgery	1.25	5.81	0.83	−10.17	12.68
	Obstetrics and gynecology	3.49	6.13	0.57	−8.57	15.55
	Emergency	−0.08	6.18	0.99	−12.24	12.07
	ICU	7.66	6.13	0.21	−4.40	19.72
Emergency	Internal medicine	−4.20	3.63	0.25	−11.35	2.94
	Surgery	1.34	3.74	0.72	−6.02	8.70
	Obstetrics and gynecology	3.58	4.22	0.40	−4.73	11.89
	Pediatric	0.08	6.18	0.99	−12.07	12.24
	ICU	7.75	4.22	0.07	−0.56	16.06
ICU	Internal medicine	−11.95^*^	3.55	0.00	−18.94	−4.97
	Surgery	−6.41	3.66	0.08	−13.61	0.79
	Obstetrics and gynecology	−4.17	4.15	0.32	−12.34	4.00
	Pediatric	−7.66	6.13	0.21	−19.72	4.40
	Emergency	−7.75	4.22	0.07	−16.06	0.56

* The mean difference is significant at the 0.05 level.

### Correlation between psychological capital and humanistic care ability of nurses

It can be seen from Table 7 that each sub-dimension and total psychological capital scores were positively correlated with humanistic care ability.

**Table 7:** Correlation analysis of psychological capital and humanistic care ability of nurses.

Item	Humanistic care ability	
	*r*	*p*
Self-efficacy	0.45	0.00
Hope	0.37	0.00
Resilience	0.37	0.00
Optimism	0.39	0.00
Psychological capital total score	0.45	0.00

We performed a stepwise multiple regression analysis using sex, age, professional title, departments, education, years of employment, growth environment, marital status, efficacy, hope, resilience, and optimism as independent variables and the humanistic care ability total score as dependent variables. The analysis showed that efficiency and optimism had statistically significant effects on humanistic care ability scores. Both of them explained 21.1% of the total variation in humanistic care ability. The standardized regression coefficient of efficiency was the largest, which was the major predictive variable affecting the humanistic care ability of nurses (Table 8).

**Table 8:** Humanistic care ability stepwise multiple regression analysis.

Model		Unstandardized coefficients		Standardized coefficients	*t*	*p*
		*B*	Std. error	Beta		
1	(Constant)	136.02	5.90		23.05	0.00
	Self-efficacy	1.94	0.21	0.45	9.12	0.00
2	(Constant)	130.60	6.17		21.18	0.00
	Self-efficacy	1.47	0.27	0.34	5.37	0.00
	Optimism	1.29	0.47	0.17	2.76	0.01

*R^2^* = 0.216, adjust *R^2^* = 0.211, *F *= 46.21, *p < *0.00.

## Discussion

### Factors influencing psychological capital of nurses

Psychological capital refers to a positive psychological state that can be developed by individuals (Avey et al., 2010) and is characterized by development and change. Professional titles, departments, years of employment, and marital status had an impact on the psychological capital of nurses.

### Professional title

Nurses with senior professional titles had the highest psychological capital scores. Senior nurses are more inclined to have a positive coping style (Shan et al., 2021). Moreover, senior nurses were generally well-qualified with extensive work experience and enthusiasm for their job, and showed strong resilience in dealing with frustration and pressure (Zhu et al., 2021). Additionally, senior nurses had mastered more professional skills and were able to manage clinical events more easily.

### Department

Nurses from obstetrics and gynecology, pediatric, and ICU had lower psychological capital scores. Obstetrics and gynecology is a high-risk technical environment in which nurses are responsible for the safety and health of women and newborns (Ribeliene et al., 2019). Studies have shown that nurses in obstetrics and gynecology, especially midwives and neonatology nurses, have high levels of anxiety, burnout and secondary traumatic stress disorder compared with nurses in other departments (De la Fuente-Solana et al., 2019). High workload and ethically challenging duties are major problems for obstetric nurses (Holmlund et al., 2022). Feelings of powerlessness, work pressure, and work frustration among obstetrics and gynecology nurses are common in the work environment (Jin et al., 2022). Investigations have shown that obstetric nurses have psychological problems such as fear and hypochondria under the COVID-19 epidemic (Wu et al., 2021). Pediatric nurses are in a similar situation to obstetric nurses. Work-related stressors cause emotional burden, psychological distress, and burnout (Macintyre et al., 2022). All these factors may affect the psychological capital of nurses.

The ICU is a complex and stressful work environment due to the critical nature of hospitalized patients, the highly technical devices and equipment used in the ICU, and the need for speedy action of nurses in inpatient care (Mehri et al., 2022). Thus, ICU nurses need to withstand a heightened risk of infection and often need to render emergency treatment within a few seconds, which is likely to cause tension (Li et al., 2021). ICUs can be stressful due to high mortality rates, critical medical conditions, and ethical dilemmas (Oliveira et al., 2019). ICU nurses are more prone to burnout syndrome because of the heavy workload and long shifts (Choudhary et al., 2022). During the COVID-19 pandemic, ICU nurses had been under great pressure (Wang et al., 2020). ICU nurses work under great physical and mental pressure, thus tend to have negative self-evaluations and the psychological capital scores are decreased.

### Years of employment

Nurses with more professional experience had higher psychological capital levels, which is consistent with the results of other studies in the literature (Jeong and Jung, 2018; Kaya and Eskin Bacaksiz, 2021). The nurses with low seniority had relatively low psychological capital scores. Transition into the workplace causes a range of stress for new graduate nurses who experience both psychological well- and ill-being (Jarden et al., 2021). Newly qualified nurses encounter multiple work-related stress over the first 12 months post-qualification (Halpin et al., 2017). Some newly qualified nurses feel overwhelmed and vulnerable. The post-registration period can be a challenging time for nurses (Collard et al., 2020). With the increase in years of employment, skills and experience would be richer than before, and psychological capital was also significantly improved.

### Marital status

The psychological capital of married nurses was higher than unmarried nurses. Functional social support could protect individuals from psychological problems by buffering the negative effect of life stressors on mental health and promoting wellbeing (Azanedo et al., 2021). The spiritual orientation of nurses has a positive effect on psychological capital. Married nurses are more spiritual than single nurses to shape empathetic behaviors (Allahyari Bouzanjani et al., 2021). Married nurses are better able to cope with stress and frustration from life and work.

### Factor influencing humanistic care ability of nurses

Humanistic caring ability is not innate, but is progressively formed and developed through one’s own learning and social practice under the interaction of environment and education (Huang et al., 2008). Studies have shown that nurses’ educational background can affect humanistic care (Mohamadi Asl et al., 2022). However, in our study, the educational background does not play a prominent role. This result is consistent with another study (Deng et al., 2019). In the past decade, nursing education in China has developed rapidly (You et al., 2015). Because of the effect of working experience, there was no difference in humanistic care ability between senior college nurses and junior undergraduate nurses.

In our study, results showed that gender was not the influencing factor of humanistic care ability, which is consistent with the study by Lai et al. (2022). But other studies showed that female nurses have higher humanistic care ability scores than males (Fei et al., 2017). This result may be due to the uneven sample size of males and females.

Results showed that the humanistic care ability of married nurses was stronger than that of single nurses. This has also been shown in other studies (Zhou et al., 2014). Empathy is the essence of communication, and effective communication promotes the development of humanistic care among nurses (Babaii et al., 2021). Life satisfaction and optimal communication within the family have a positive effect on relationship satisfaction (Sztanyi-Szeker et al., 2022), which helps nurses effectively carry out humanistic care.

### Relationship between nurses’ psychological capital and humanistic care ability

Psychological capital was positively correlated with humanistic care ability. A high level of psychological capital enhances an individuals’ active engagement in work (Setar et al., 2015). Members with greater psychological capital try harder with the conviction that they can achieve better performance (self-efficacy), derive multiple ways to solve problems with strong willpower (hope), expect positive results based on internal attribution (optimism), and make efforts to respond positively to difficult situations [resilience] (Jang, 2022). Psychological capital has a significant impact on lower rates of burnout and turnover (Lee et al., 2019). Nurses with high psychological capital have more enthusiasm to care for patients.

Self-efficiency and optimism directly affect humanistic care ability. Self-efficacy is significantly associated with work-related stress and mental health problems (Azemi et al., 2022). Self-efficacy plays a significant role in understanding consequences of occupational stress (Dianat et al., 2021). The level of self-efficacy determines the success rate of the event (Herts et al., 2017). Nurses with low self-efficacy have stress and anxiety during difficult situations, which in turn hamper work (Xiong et al., 2020). High self-efficacy which ensures proactive work and better goal achievement (Lim et al., 2022) could improve the quality of care and nurse–patient relationships (Bu et al., 2021).

Optimism is a positive emotion. A growing body of evidence highlights the unique, independent role of positive emotion in promoting adaptive coping in the face of stress (Cheung et al., 2020). From an optimist’s point of view, negative events are transient, of an external cause, and specific to a given fact, and the positive events are linked to internal issues that are more permanent and recurrent (Almeida and Miclos, 2022). Optimists are more likely to provide humanistic care to patients to cope with a disease positively.

### Practical implications

By identifying the relationship between psychological capital and humanistic caring capacity, our study offers some practical implications.

We suggest that nursing managers can improve the humanistic care ability of nurses by reinforcing their self-efficacy. High self-efficacy can help nurses show greater confidence in their work and develop positive relationships with patients.

Nursing managers can also implement psychological interventions to keep nurses optimistic so that nurses are more willing to practice humanistic care.

Nowadays, positive psychology has been widely used. Positive psychology is regarded as an academic field encompassing character strengths, positive relationships, experience, and institutions (Seligman, 2019). Short-term positive psychological coaching is a valuable method for developing personal psychological capital (Corbu et al., 2021). Positive psychological group intervention effectively enhanced positive emotions (Kounenou et al., 2022). Positive psychological intervention will play a greater role in improving humanistic care ability.

### Limitations

Several limitations need to be considered. First, the nurse participants were conveniently recruited from one hospital, resulting in findings that may not represent all Chinese nurses. Second, self-report measures were employed, which may affect the objectivity and authenticity of the collected data. Third, only psychological capital and some socio-demographic characteristics were tested as influences on humanistic caring ability; other possible determinants need to be explored for comprehensively understanding humanistic caring ability. Longitudinal research designs and randomized sampling are recommended in future studies.

## Conclusion

Psychological capital positively affected the humanistic care ability of nurses. Self-efficacy was the main predictor of humanistic care ability. Nursing managers can formulate strategies from the perspective of positive psychology to improve humanistic care ability of nurses.

## Data availability statement

The original contributions presented in the study are included in the article/supplementary material, further inquiries can be directed to the corresponding author.

## Ethics statement

Ethical review and approval were not required for the study on human participants in accordance with the local legislation and institutional requirements. The participants provided their written informed consent to participate in this study.

## Author contributions

XL conceived and designed the study and reviewed the manuscript. XL and XY collected the data. XL and CL interpreted the data. XL wrote the first draft of the manuscript. CL and BS modified the manuscript. All authors contributed to the article and approved the submitted version.

## Conflict of interest

The authors declare that the research was conducted in the absence of any commercial or financial relationships that could be construed as a potential conflict of interest.

## Publisher’s note

All claims expressed in this article are solely those of the authors and do not necessarily represent those of their affiliated organizations, or those of the publisher, the editors and the reviewers. Any product that may be evaluated in this article, or claim that may be made by its manufacturer, is not guaranteed or endorsed by the publisher.

## References

[ref1] Allahyari Bouzanjani A. Bahadori P. Nikoonam P. (2021). Nurses' empathetic behaviors: the direct and indirect effect of their spiritual orientation. J. Relig. Health 60, 134–152. doi: 10.1007/s10943-019-00966-9, PMID: 31894520

[ref2] Almeida D. Miclos P. V. (2022). Nursing in primary health care: association between leadership, psychological capital, and burnout implications. Rev. Bras. Enferm. 75:e20210942. doi: 10.1590/0034-7167-2021-0942, PMID: 35858010

[ref3] An M. Shin E. S. Choi M. Y. Lee Y. Hwang Y. Y. Kim M. (2020). Positive psychological capital mediates the association between burnout and nursing performance outcomes among hospital nurses. Int. J. Environ. Res. Public Health 17:5988. doi: 10.3390/ijerph17165988, PMID: 32824724 PMC7460099

[ref4] Antonini M. Bellier-Teichmann T. O’Reilly L. Cara C. Brousseau S. Weidmann J.. (2021). Effects of an educational intervention to strengthen humanistic practice on haemodialysis nurses’ caring attitudes and behaviours and quality of working life: A cluster randomised controlled trial. BMC Nurs. 20:255. doi: 10.1186/s12912-021-00729-6, PMID: 34930206 PMC8691052

[ref5] Avey J. B. Luthans F. Smith R. M. Palmer N. F. (2010). Impact of positive psychological capital on employee well-being over time. J. Occup. Health Psychol. 15, 17–28. doi: 10.1037/a0016998, PMID: 20063956

[ref6] Azanedo C. M. Artola T. Sastre S. Alvarado J. M. (2021). Character strengths predict subjective well-being, psychological well-being, and psychopathological symptoms, over and above functional social support. Front. Psychol. 12:661278. doi: 10.3389/fpsyg.2021.661278, PMID: 34621205 PMC8490684

[ref7] Azemi S. Dianat I. Abdollahzade F. Bazazan A. Afshari D. (2022). Work-related stress, self-efficacy and mental health of hospital nurses. Work 72, 1007–1014. doi: 10.3233/WOR-210264, PMID: 35634826

[ref8] Babaii A. Mohammadi E. Sadooghiasl A. (2021). The meaning of the empathetic nurse-patient communication: a qualitative study. J. Patient Exp. 8:23743735211056432. doi: 10.1177/23743735211056432, PMID: 34869836 PMC8640307

[ref9] Bu M. Ma H. Zhai H. Ma Y. Xu N. (2021). Role of self-efficacy in nursing organizational climate: a way to develop nurses' humanistic practice ability. J. Nurs. Manag. doi: 10.1111/jonm.13516, PMID: 34798681

[ref10] Calle G. H. Martin M. C. Nin N. (2017). Seeking to humanize intensive care. Rev. Bras. Ter. Intensiva 29, 9–13. doi: 10.5935/0103-507X.20170003, PMID: 28444067 PMC5385980

[ref11] Cheung E. O. Hernandez A. Herold E. Moskowitz J. T. (2020). Positive emotion skills intervention to address burnout in critical care nurses. AACN Adv. Crit. Care 31, 167–178. doi: 10.4037/aacnacc2020287, PMID: 32526000

[ref12] Choudhary M. Kumar A. Pandey V. Choudhary V. Patidar N. (2022). Burnout syndrome assessment scale for nurses working in intensive care units: development and validation. Int. J. Appl. Basic Med. Res. 12, 82–86. doi: 10.4103/ijabmr.ijabmr_547_21, PMID: 35754669 PMC9215192

[ref13] Collard S. S. Scammell J. Tee S. (2020). Closing the gap on nurse retention: a scoping review of implications for undergraduate education. Nurse Educ. Today 84:104253. doi: 10.1016/j.nedt.2019.104253, PMID: 31706205

[ref14] Corbu A. Pelaez Zuberbuhler M. J. Salanova M. (2021). Positive psychology micro-coaching intervention: effects on psychological capital and goal-related self-efficacy. Front. Psychol. 12:566293. doi: 10.3389/fpsyg.2021.566293, PMID: 33643121 PMC7904700

[ref15] De la Fuente-Solana E. I. Suleiman-Martos N. Pradas-Hernandez L. Gomez-Urquiza J. L. Canadas-De la Fuente G. A. Albendin-Garcia L. (2019). Prevalence, related factors, and levels of burnout syndrome among nurses working in gynecology and obstetrics services: a systematic review and meta-analysis. Int. J. Environ. Res. Public Health 16:2585. doi: 10.3390/ijerph16142585, PMID: 31331046 PMC6678444

[ref16] Deng J. Lei L. Zhang H. L. Luo Y. (2019). The current status and the influencing factors of humanistic care ability among a group of medical professionals in Western China. Technol. Health Care 27, 195–208. doi: 10.3233/THC-181389, PMID: 30562911 PMC6484270

[ref17] Dianat I. Azemi S. Abdollahazade F. Bazazan A. Asghari Jafarabadi M. (2021). Does self-efficacy mediate the relationship between occupational stress and mental health problems? A study among nursing professionals. Health Promot. Perspect. 11, 344–350. doi: 10.34172/hpp.2021.44, PMID: 34660230 PMC8501481

[ref18] Dwyer P. A. Hunter Revell S. M. Sethares K. A. Ayotte B. J. (2019). The influence of psychological capital, authentic leadership in preceptors, and structural empowerment on new graduate nurse burnout and turnover intent. Appl. Nurs. Res. 48, 37–44. doi: 10.1016/j.apnr.2019.04.005, PMID: 31266606

[ref19] Elliott R. Fry M. (2021). Psychological capital, well-being, and patient safety attitudes of nurses and midwives: a cross-sectional survey. Nurs. Health Sci. 23, 237–244. doi: 10.1111/nhs.12808, PMID: 33382147

[ref20] Fei D. Sheng L. Peng D. Peng K. Zhao L. (2017). Research progress on the current situation and influencing factors of humanistic care ability of nurses in China. Chin. Gen. Pract. Nurs. 15, 1429–1432. doi: 10.3969/j.issn.1674-4748.2017.012.007

[ref21] Gao M. Wang Y. Lei Y. Zhang L. Li L. Wang C.. (2021). Applying the Carolina care model to improve nurses' humanistic care abilities. Am. J. Transl. Res. 13, 3591–3599. 34017540 PMC8129219

[ref22] Goral Turkcu S. Ozkan S. (2021). The effects of reflexology on anxiety, depression and quality of life in patients with gynecological cancers with reference to Watson's theory of human caring. Complement. Ther. Clin. Pract. 44:101428. doi: 10.1016/j.ctcp.2021.101428, PMID: 34157494

[ref23] Guo Y. F. Cross W. M. Lam L. Plummer V. Wang X. X. Wang S. S. (2021). Association between psychological capital and spiritual care competencies of clinical nurses: a multicentre cross-sectional study. J. Nurs. Manag. 29, 1713–1722. doi: 10.1111/jonm.13303, PMID: 33682206

[ref24] Halpin Y. Terry L. M. Curzio J. (2017). A longitudinal, mixed methods investigation of newly qualified nurses' workplace stressors and stress experiences during transition. J. Adv. Nurs. 73, 2577–2586. doi: 10.1111/jan.13344, PMID: 28543602

[ref25] Herts K. L. Khaled M. M. Stanton A. L. (2017). Correlates of self-efficacy for disease management in adolescent/young adult cancer survivors: A systematic review. Health Psychol. 36, 192–205. doi: 10.1037/hea0000446, PMID: 27929332

[ref26] Holmlund S. Lan P. T. Edvardsson K. Ntaganira J. Graner S. Small R.. (2022). Vietnamese midwives' experiences of working in maternity care: A qualitative study in the Hanoi region. Sex. Reprod. Healthc. 31:100695. doi: 10.1016/j.srhc.2022.100695, PMID: 35085930

[ref27] Huang Y. Xu L. Jiang X. (2008). A survey of status quo of humanistic caring ability of nursing students. Chin. Nurs. Res. 22, 673–675.

[ref28] Jang E. (2022). Authentic leadership and task performance via psychological capital: the moderated mediation role of performance pressure. Front. Psychol. 13:722214. doi: 10.3389/fpsyg.2022.722214, PMID: 35712172 PMC9197480

[ref29] Jarden R. J. Jarden A. Weiland T. J. Taylor G. Bujalka H. Brockenshire N.. (2021). New graduate nurse wellbeing, work wellbeing and mental health: a quantitative systematic review. Int. J. Nurs. Stud. 121:103997. doi: 10.1016/j.ijnurstu.2021.103997, PMID: 34218048

[ref30] Jeong E. Jung M.-R. (2018). Influences of compassion satisfaction, compassion fatigue, and burnout on positive psychological capital of clinical nurses. J. Korea Contents Assoc. 18, 246–255. doi: 10.5392/JKCA.2018.18.03.246

[ref31] Jiang T. Tang P. Li Y. Shi T. (2017). Relationship among humanistic care ability, positive psychological capital and subjective well-being in undergraduate nursing students. Med. Philosophy 38, 39–41. doi: 10.12014/j.issn.1002-0772.2017.08a.13

[ref32] Jin Y. Bi Q. Song G. Wu J. Ding H. (2022). Psychological coherence, inclusive leadership and implicit absenteeism in obstetrics and gynecology nurses: a multi-site survey. BMC Psychiatry 22:525. doi: 10.1186/s12888-022-04137-1, PMID: 35922834 PMC9351111

[ref33] Kaya G. Eskin Bacaksiz F. (2021). The relationships between nurses' positive psychological capital, and their employee voice and organizational silence behaviors. Perspect. Psychiatr. Care. doi: 10.1111/ppc.12990, PMID: 34888883

[ref34] Kim M. (2022). The factors associated with person-centered care competence among nursing students. Int. J. Environ. Res. Public Health 19:2787. doi: 10.3390/ijerph19052787, PMID: 35270478 PMC8910480

[ref35] Kounenou K. Kalamatianos A. Garipi A. Kourmousi N. (2022). A positive psychology group intervention in Greek university students by the Counselling Centre: effectiveness of implementation. Front. Psychol. 13:965945. doi: 10.3389/fpsyg.2022.965945 36092081 PMC9450998

[ref36] Kovner C. Brewer C. Wu Y. W. Cheng Y. Suzuki M. (2006). Factors associated with work satisfaction of registered nurses. J. Nurs. Scholarsh. 38, 71–79. doi: 10.1111/j.1547-5069.2006.00080.x 16579327

[ref37] Lai L. Ding S. Zhong Z. Mao P. Sun N. Zheng F. (2022). Association between positive mental character and humanistic care ability in Chinese nursing students in Changsha, China. Front. Psychol. 13:896415. doi: 10.3389/fpsyg.2022.896415, PMID: 35795450 PMC9251419

[ref38] Lee H. F. Chiang H. Y. Kuo H. T. (2019). Relationship between authentic leadership and nurses' intent to leave: the mediating role of work environment and burnout. J. Nurs. Manag. 27, 52–65. doi: 10.1111/jonm.12648, PMID: 30238670

[ref39] Letourneau D. Goudreau J. Cara C. (2021). Humanistic caring, a nursing competency: modelling a metamorphosis from students to accomplished nurses. Scand. J. Caring Sci. 35, 196–207. doi: 10.1111/scs.12834, PMID: 32141649

[ref40] Li S. Chong E. S. Peng C. Zhang H. (2017). Implimenting the "humanistic care model"in the undergraduate nursing education. DEStech Trans. Econ. Bus., 237–243. doi: 10.12783/dtem/emem2017/17089

[ref41] Li J. Zhang Y. Li L. Yi W. Hao Y. Bi Y. (2021). Predictive analysis of factors influencing depression status of nurses in the COVID-19 pandemic intensive care unit. Front. Psych. 12:596428. doi: 10.3389/fpsyt.2021.596428, PMID: 34867493 PMC8636193

[ref42] Lim S. Song Y. Nam Y. Lee Y. Kim D. (2022). Moderating effect of burnout on the relationship between self-efficacy and job performance among psychiatric nurses for COVID-19 in national hospitals. Medicina 58:171. doi: 10.3390/medicina58020171, PMID: 35208495 PMC8880477

[ref43] Luo H. He Z. (2010). Reliability and validity of psychological capital questionnaire in nurses Chinese journal of behavioral medicine and brain. Science 19, 853–854. doi: 10.3760/cma.J

[ref44] Luthans F. Avolio B. J. Avey J. B. Norman S. M. (2007a). Positive psychological capital: measurement and relationship with performance and satisfaction. Pers. Psychol. 60, 541–572. doi: 10.1111/j.1744-6570.2007.00083.x

[ref45] Luthans F. Luthans K. W. Luthans B. C. (2004). Positive psychological capital: beyond human and social capital. Bus. Horiz. 47, 45–50. doi: 10.1016/j.bushor.2003.11.007

[ref46] Luthans F. Youssef C. M. Avolio B. J. (2007b). *Psychological* Capital: Developing the Human Competitive Edge. Oxford: Oxford University Press.

[ref47] Luthans F. Youssef-Morgan C. M. (2017). Psychological capital: An evidence-based positive approach. Annu. Rev. Organ. Psych. Organ. Behav. 4, 339–366. doi: 10.1146/annurev-orgpsych-032516-113324

[ref48] Ma J. Peng W. Pan J. (2022). Investigation into the correlation between humanistic care ability and emotional intelligence of hospital staff. BMC Health Serv. Res. 22:839. doi: 10.1186/s12913-022-08227-4, PMID: 35773661 PMC9244559

[ref49] Macintyre M. R. Brown B. W. J. Schults J. A. (2022). Factors influencing pediatric hematology/oncology nurse retention: a scoping review. J. Pediatr. Hematol. Oncol. Nurs. doi: 10.1177/27527530221099899 [Epub ahead of print]., PMID: 35815893

[ref50] McCance T. V. McKenna H. P. Boore J. R. (1999). Caring: theoretical perspectives of relevance to nursing. J. Adv. Nurs. 30, 1388–1395. doi: 10.1046/j.1365-2648.1999.01214.x, PMID: 10583650

[ref51] Mehri F. Babaei-Pouya A. Karimollahi M. (2022). Intensive care unit nurses in Iran: occupational cognitive failures and job content. Front. Public Health 10:786470. doi: 10.3389/fpubh.2022.786470, PMID: 35570927 PMC9099024

[ref52] Mohamadi Asl S. Khademi M. Mohammadi E. (2022). The influential factors in humanistic critical care nursing. Nurs. Ethics 29, 608–620. doi: 10.1177/09697330211043274, PMID: 35144499

[ref53] Nkongho N. O. (1990). The Caring Ability Inventory. New York: Springer Publishing Co.

[ref54] Oliveira E. G. Garcia P. C. Citolino Filho C. M. de Souza Nogueira L. (2019). The influence of delayed admission to intensive care unit on mortality and nursing workload: a cohort study. Nurs. Crit. Care 24, 381–386. doi: 10.1111/nicc.12402, PMID: 30478867

[ref55] Rafii F. Nasrabadi A. N. Tehrani F. J. (2021). The omission of some patterns of knowing in clinical care: a qualitative study. Iran. J. Nurs. Midwifery Res. 26, 508–514. doi: 10.4103/ijnmr.IJNMR_75_20, PMID: 34900649 PMC8607894

[ref56] Raja S. Hasnain M. Vadakumchery T. Hamad J. Shah R. Hoersch M. (2015). Identifying elements of patient-centered care in underserved populations: a qualitative study of patient perspectives. PLoS One 10:e0126708. doi: 10.1371/journal.pone.0126708, PMID: 25993110 PMC4437903

[ref57] Ribeliene J. Blazeviciene A. Nadisauskiene R. J. Tameliene R. Kudreviciene A. Nedzelskiene I.. (2019). Patient safety culture in obstetrics and gynecology and neonatology units: the nurses' and the midwives' opinion. J. Matern. Fetal Neonatal Med. 32, 3244–3250. doi: 10.1080/14767058.2018.1461831, PMID: 29618234

[ref58] Seligman M. E. P. (2019). Positive psychology: a personal history. Annu. Rev. Clin. Psychol. 15, 1–23. doi: 10.1146/annurev-clinpsy-050718-095653 30525996

[ref59] Setar S. Buitendach J. Kanengoni H. (2015). The moderating role of psychological capital in the relationship between job stress and the outcomes of incivility and job involvement amongst call Centre employees. SA J. Ind. Psychol. 41, 1–13. doi: 10.4102/sajip.v41i1.1183

[ref60] Shan Y. Shang J. Yan Y. Lu G. Hu D. Ye X. (2021). Mental workload of frontline nurses aiding in the COVID-19 pandemic: a latent profile analysis. J. Adv. Nurs. 77, 2374–2385. doi: 10.1111/jan.14769, PMID: 33594687 PMC8014576

[ref61] Shen M. Zhao X. Ye H. Zhang X. Fang J. (2020). Correlation between individual social capital and humanistic care ability in nursing students: multiple mediating role of positive psychological capital and adaptive emotion expression. Chin. J. Mod. Nurs. 26, 293–298. doi: 10.3760/cma.j.issn.1674-2907.2020.03.003

[ref62] Sheng L. Wang J. Li X. Fei D. Zhao L. (2018). Survey on current situation of nurse’ humanistic care ability and the relationship with head nurse’s leadership. J. Nurs. Adm. 18, 316–319. doi: 10.3969/j.issn.1671-315x.2018.05.004

[ref63] Smith M. C. Parker M. E. (2015). Nursing Theories and Nursing Practice. (4th Edn.). Philadelphia: F.A. Davis Company.

[ref64] Sztanyi-Szeker B. Hogye-Nagy A. Szeman-Nagy A. (2022). Connection between satisfaction with relationship-, life, well-being and the development of the family structure. Psychiatr. Hung. 37, 124–132. 35582866

[ref65] Wang L. Xiang Y. Shao Q. Song J. Jin Q. Ye W. (2020). Investigations on mental health of ICU nurses supported in Wuhan. Chin. J. Nurs. 55, 129–131. doi: 10.3761/j.issn.0254-1769.2020.S1.045

[ref66] Wang Y. Zhang Y. Liu M. Zhou L. Zhang J. Tao H.. (2020). Research on the formation of humanistic care ability in nursing students: a structural equation approach. Nurse Educ. Today 86:104315. doi: 10.1016/j.nedt.2019.104315, PMID: 31896034

[ref67] Watson J. (2002). Assessing and Measuring Caring in Nursing and Health Science. New York: Springer Publishing Company Inc.

[ref68] Woods M. (2011). An ethic of care in nursing: past, present and future considerations. Ethic. Soc. Welfare 5, 266–276. doi: 10.1080/17496535.2011.563427

[ref69] Wu Y. Han B. Xu F. Guo L. Liu X. Zheng S. (2021). Correlation analysis between psychological stress and coping style of obstetrics nurses in the case of COVID-19. Chin. Nurs. Manag. 21, 219–223. doi: 10.3969/j.issn.1672-1756.2021.02.014

[ref70] Xiong H. Yi S. Lin Y. (2020). The psychological status and self-efficacy of nurses during COVID-19 outbreak: a cross-sectional survey. Inquiry 57, 1–16. doi: 10.1177/0046958020957114, PMID: 32900271 PMC7485150

[ref71] Yan Y. Zhao S. Li Y. Luo M. Liu S. (2022). Mediating effect analysis of professional value of standardized training on humanistic care cognition and humanistic care ability. Acta Med. Mediterranea 38, 1787–1792. doi: 10.19193/0393-6384_2022_3_273

[ref72] Yanmis S. Bahcecioglu Turan G. Ozer Z. (2022). Turkish validity and reliability study of humanistic practice ability of nursing scale. Int. J. Clin. Pract. 2022, 8435530–8435538. doi: 10.1155/2022/8435530, PMID: 35685539 PMC9159197

[ref73] Yeh M. Y. Lee S. (2011). The spirit of humanism should be cultivated in the nursing profession. Hu Li Za Zhi 58, 12–16. PMID: 22024799

[ref74] You L. M. Ke Y. Y. Zheng J. Wan L. H. (2015). The development and issues of nursing education in China: a national data analysis. Nurse Educ. Today 35, 310–314. doi: 10.1016/j.nedt.2014.10.004, PMID: 25456256

[ref75] Zhou L. Gao L. Xu G. Ye P. (2014). Investigation and analysis of nursing humanistic caring capability. J. Benbu Med. Coll. 39, 823–825; 828. doi: 10.13898/j.cnki.issn.1000-2200.2014.06.017

[ref76] Zhou J. Yang Y. Qiu X. Yang X. Pan H. Ban B.. (2018). Serial multiple mediation of organizational commitment and job burnout in the relationship between psychological capital and anxiety in Chinese female nurses: A cross-sectional questionnaire survey. Int. J. Nurs. Stud. 83, 75–82. doi: 10.1016/j.ijnurstu.2018.03.016, PMID: 29702357

[ref77] Zhu X. Shen L. Du P. Guan J. (2021). The mediating role of psychological capital between Dayadi stress and job burnout in female nurses with two children. Trans. Pediatr. 10, 2449–2458. doi: 10.21037/tp-21-375, PMID: 34765468 PMC8578747

